# Acute Effect of Enhanced External Counterpulsation on the Carotid Hemodynamic Parameters in Patients With High Cardiovascular Risk Factors

**DOI:** 10.3389/fphys.2021.615443

**Published:** 2021-06-17

**Authors:** Yahui Zhang, Zhouming Mai, Jianhang Du, Wenjuan Zhou, Wenbin Wei, Hui Wang, Chun Yao, Xinxia Zhang, Hui Huang, Guifu Wu

**Affiliations:** ^1^Department of Cardiology, The Eighth Affiliated Hospital of Sun Yat-sen University, Shenzhen, China; ^2^National Health Commission (NHC) Key Laboratory of Assisted Circulation, Sun Yat-sen University, Guangzhou, China; ^3^Guangdong Innovative Engineering and Technology Research Center for Assisted Circulation, Shenzhen, China; ^4^Department of Cardiac Ultrasound, The Eighth Affiliated Hospital, Sun Yat-sen University, Shenzhen, China

**Keywords:** enhanced external counterpulsation, hemodynamic responses, cardiovascular risks, Doppler ultrasound images, common carotid artery

## Abstract

**Purpose:**

Enhanced external counterpulsation (EECP) can improve carotid circulation in patients with coronary artery disease. However, the response of carotid hemodynamic parameters induced by EECP in patients with high cardiovascular risk factors remains to be clarified. This study aimed to investigate the acute effect of EECP on the hemodynamic parameters in the carotid arteries before, during, and immediately after EECP in patients with hypertension, hyperlipidemia, and type 2 diabetes.

**Methods:**

Eighty-three subjects were recruited into this study to receive 45-min EECP, including patients with simple hypertension (*n* = 21), hyperlipidemia (*n* = 23), type 2 diabetes (*n* = 18), and healthy subjects (*n* = 21). Hemodynamic parameters in both common carotid arteries (CCAs) were measured and calculated from Doppler ultrasound images. Peak systolic velocity (PSV), end-diastolic velocity (EDV), mean inner diameter (ID), systolic/diastolic flow velocity ratio (VS/VD), flow rate (FR), and resistance index (RI) were monitored before, during, and immediately after 45-min EECP.

**Results:**

EDV and VS/VD were significantly reduced, while RI of CCAs was significantly increased among four groups during EECP (all *P* < 0.01). Additionally, the ID of CCAs and the FR of left CCA increased in patients with hyperlipidemia during EECP (*P* < 0.05). PSV of left CCA was reduced in patients with type 2 diabetes (*P* < 0.05). Moreover, immediately after EECP, ID was significantly higher in patients with hyperlipidemia. The RI of patients with hypertension and PSV and VS/VD of patients with type 2 diabetes were significantly lower compared with baseline (all *P* < 0.05).

**Conclusion:**

EECP created an acute reduction in EDV, PSV, and VS/VD, and an immediate increase in the RI, FR, and ID of CCAs among the four groups. Additionally, a single 45-min session of EECP produced immediate improvement in the ID of patients with hyperlipidemia, the RI of patients with hypertension, and the PSV and VS/VD of patients with type 2 diabetes. The different hemodynamic responses induced by EECP may provide theoretical guidance for making personalized plans in patients with different cardiovascular risk factors.

## Introduction

Enhanced external counterpulsation (EECP) is a non-invasive FDA-approved treatment that reduces angina and improves myocardial ischemia in patients with coronary heart disease (CHD) (Masuda et al., 2001; Gloekler et al., 2010). EECP includes sequential inflation and deflation of compressible cuffs wrapped around the participant’s calves, lower thighs, and upper thighs. Compressed air pressure is used by the cuffs to the lower extremities in a sequence synchronized with the cardiac cycle *via* identifying ECG signals. Studies have also reported that EECP was beneficial for peripheral vascular function (e.g., blood pressure (BP) and blood flow) in patients with CHD (Michaels, 2002; Braith et al., 2010). However, the effects of EECP on cardiovascular function in patients with different cardiovascular risk factors, such as hypertension, hyperlipidemia, and type 2 diabetes, remains controversial.

Bondesson et al. (2010) found that EECP treatment decreased BP in patients with refractory angina pectoris. By contrast, Lin et al. (2012) found that EECP significantly increased the mean BP of stroke patients (12.84%). Sardina et al. (2016) reported that EECP decreased advanced glycation end product (AGE/RAGE) concentrations, oxidative stress, and inflammation in patients with type II diabetes mellitus, but it is unclear how EECP will influence those parameters. Martin et al. (2013) found that the benefits of EECP therapy in people with abnormal glucose tolerance may contribute to microvascular function. Other studies showed that EECP improved vascular endothelial function and wave reflection characteristics and reduced arterial stiffness (Nichols et al., 2006), while a study found that EECP cannot reduce arterial stiffness (Dockery et al., 2004).

Acute intervention is an effective method of investigating the peripheral hemodynamic parameter responses to external stimuli (Liu et al., 2015; Zhang et al., 2018). Michaels (2002) found that average peak velocity significantly increased during EECP, and coronary flow showed a 28% increase during EECP compared with baseline. Gurovich and Braith (2013) reported that EECP acutely improves endothelium-dependent vasodilation in both femoral and brachial arteries in young people (Gurovich and Braith, 2013). Levenson et al. (2007) showed that EECP exerts vascular relaxation effects on both large and small arteries of the carotid circulation in patients with coronary artery disease immediately after EECP. To date, however, little attention has been paid to the acute impact of EECP on carotid vascular function in patients with high cardiovascular risk factors.

The responses of peak systolic velocity (PSV), resistance index (RI), mean inner diameter (ID), end-diastolic velocity (EDV), and blood flow in the left and right common carotid arteries (CCAs) may exhibit different prognostic values in the investigated population (Lee et al., 2011; Loizou et al., 2015) and are closely related to cardiovascular events (Bai et al., 2007; Ozari et al., 2012; Chuang et al., 2016). Kallikazaros et al. (1999) and Sedaghat et al. (2018) found that the ID of the carotid artery was closely related to CHD based on large samples. However, few studies focused on the acute effect of EECP on carotid hemodynamics. Moreover, the mechanisms of acute responses, which can highlight the responses of cardiovascular disease, have not yet been clarified (Levenson et al., 2007; Zhang et al., 2007, 2018).

This study aimed to investigate the acute effect of EECP on the hemodynamic parameters in CCAs before, during, and immediately after EECP in patients with hypertension, hyperlipidemia, and type 2 diabetes and healthy controls. Meanwhile, we try to find out sensitive hemodynamic parameters reflecting different cardiovascular risk factors and then explore the response mechanism induced by EECP.

## Materials and Methods

### Participants

Patients with simple hypertension (*n*=21), simple hyperlipidemia (*n*=23), and simple type 2 diabetes (*n*=18) were included who had enrolled in the Cardiovascular Medicine of Eighth Affiliated Hospital of Sun Yat-sen University (SYSU). In addition, healthy controls (*n*=21) were enrolled from the Health Examination Center of the Eighth Affiliated Hospital of SYSU. Before the experiment, informed consent forms were signed by all of the subjects. The study was approved by the local medical ethics committee of the Eighth Affiliated Hospital of SYSU.

### Experiment Scheme

Before this experiment, all participants were required not to ingest any food or flavonoid-containing beverages and ethanol after midnight, and to avoid alcohol, caffeine, and EECP or exercise for at least 24 h prior to the measurements. Their carotid hemodynamic data were collected in the Enhanced External Counterpulsation Center. The baseline measurements were performed for each group in the supine position after 10 min of relaxation. All subjects were received a 45-min session EECP treatment with the PSK P-ECP/TM Oxygen Saturation Monitoring EECP Instrument (made in Chongqing, China). These participants lay supine on the EECP treatment bed with their legs and buttocks wrapped in cuffs, which were sequentially inflated from the lower thigh to the upper thigh and buttocks at the beginning of the diastolic phase, followed by a quick, simultaneous deflation of all cuffs just prior to the onset of systole. The EECP treatment pressure was set as 0.028–0.033 MPa. Color Doppler Ultrasound (GE Logiq E, Universal Imaging, Wayne, NJ, United States) was used to measure at rest, 15–25 min during EECP and immediately after 45 min of EECP. The right and left CCAs were examined with 1.5 cm proximal to the bifurcation of the vessels.

### Parameter Calculation

Parameters, PSV, RI, peak diastolic velocity (VD), PSV/VD (VS/VD), and velocity-time integral (VTI) in the CCAs were continuously recorded for 10 s and then were calculated for mean value.

The mean IDs of all arteries were calculated as:

(1) $$I\,D=\left(I\,D_{d\,i\,a}+I\,D_{s\,y\,s}\right)/2;$$

where *ID*_*sys*_ and *ID*_*dia*_ are the systolic and diastolic diameters, respectively.

*RI* was analyzed only in the carotid arteries because, as a measure of cerebral resistance, it is closely associated with cardiovascular risk was calculated as:

(2) R⁢I=(P⁢S⁢V-E⁢D⁢V)/P⁢S⁢V,

where PSV is the peak systolic velocity (PSV), and EDV is the end-diastolic velocity (EDV).

Flow rate (FR) was calculated from the vessel diameter, cardiac period, and velocity-time integral as:

(3) $$F\,l\,o\,w\,r\,a\,t\,e=\left(\frac{1}{4}\,\pi \,I\,D^{2}\times V\,T\,I\right)/T,$$

where VTI is the averaged velocity-time integral, and T is the averaged cardiac cycle time.

### Statistical Analysis

ID, PSV, EDV, RI, and VS/VD are the mean values of area under the envelope curve in a cardiac cycle. Results are shown as means ± SD. Normal distribution for all the carotid hemodynamic variables was evaluated by the Kolmogorov–Smirnov test (at least one test *P* > 0.05). The difference of basic characteristics among the four groups was performed by one-way ANOVA. The repeated ANOVAs comparing carotid hemodynamic parameters before, during, and immediately after EECP and between patients with cardiovascular risks and healthy controls were performed. Fisher’s least significant difference was conducted as *post-hoc analysis.* All statistical tests were conducted by SPSS version 20.0 (IBM SPSS Statistics, Chicago, IL, United States), and *P* < 0.05 was taken as a measure of statistical significance.

## Results

Table 1 shows the base information (age, gender, height, and weight), clinical information (stroke volume (SV), left ventricle ejection fraction (LVEF), left ventricular end diastolic diameter (LVDD), blood sugar, and blood lipids), and risk factors (smoking and drinking) among the four groups. There were significant differences in blood sugar, triglyceride, total cholesterol, and glycosylated hemoglobin (*P* < 0.05), while age, gender, height, weight, SV, LVEF, LVDD, smoking, and drinking had no significant differences in each group at baseline (*P* > 0.05).

**TABLE 1:** Base characteristics, clinic information and risk factors of study population.

	**Hypertension**	**Hyperlipidemia**	**Type 2 diabetes**	**Health**	***P*-value**
Number (No.)	21	23	18	21	0.272
Age (years old)	58.86 ± 9.75	60.48 ± 7.48	60.11 ± 6.92	53.95 ± 8.44	0.196
Gender (female/%)	5 (23.8)	16 (69.6)	15 (83.3)	15 (71.4)	0.063
Height (cm)	167.1 ± 7.1	163.85 ± 6.68	158.35 ± 7.62	161.3 ± 9.5	0.196
Weight (kg)	68.93 ± 8.75	62.35 ± 9.76	58.41 ± 11.61	65.3 ± 59.24	0.311
SV (ml)	80.22 ± 15.81	71.2 ± 10.16	77 ± 16.43	64 ± 20.92	0.260
EF (%)	69.2 ± 5.53	69.8 ± 3.91	67.33 ± 8.09	67.25 ± 7.09	0.190
LVIDD (mm)	48.4 ± 4.38	46.7 ± 3.02	49.17 ± 5.85	45 ± 4.72	0.619
Blood sugar (mmol/L)	4.68 ± 0.9	4.18 ± 0.66	7.6 ± 1.05	4.17 ± 0.64	0.045
Triglyceride (mmol/L)	1.33 ± 0.39	1.75 ± 0.72	1.19 ± 0.34	1.83 ± 1.16	0.037
LDL_cholesterol (μmmol/L)	3.02 ± 0.65	3.97 ± 0.52	3.37 ± 0.78	3.09 ± 0.63	0.254
Total cholesterol (mmol/L)	4.93 ± 0.8	6.31 ± 0.49	5.34 ± 0.98	5.17 ± 0.91	0.000
Glycosylated Hemoglobin (%)	5.76 ± 0.48	5.58 ± 0.23	6.54 ± 0.79	5.57 ± 0.39	0.008
Smoking	3 (14.3)	2 (8.7)	0 (0)	0 (0)	0.153
Drinking	0 (0)	1 (4.3)	1 (5.6)	4 (19)	0.096

*SV, stroke volume; LVEF, left ventricle ejection fraction; LVDD, left ventricular end diastolic diameter.*

**FIGURE 1:**
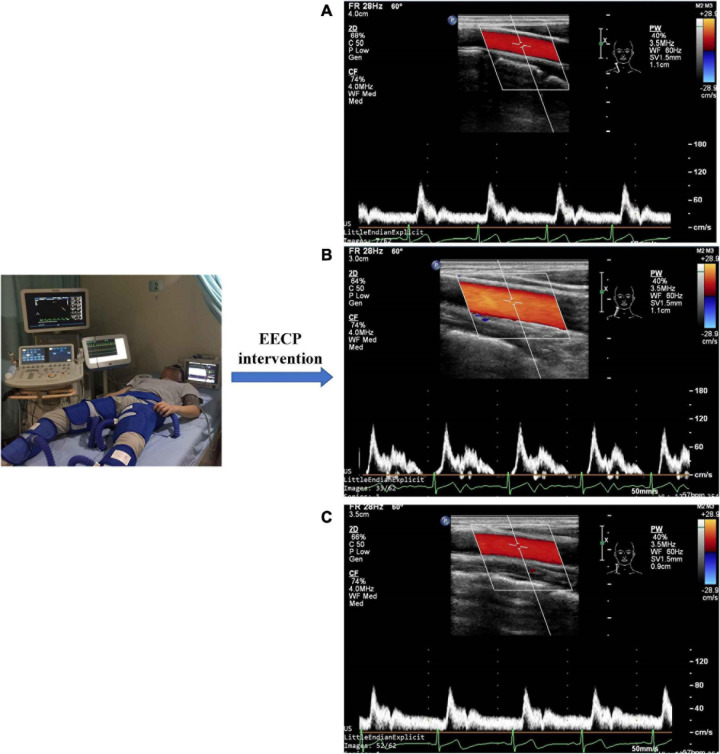
Measurement of blood flow velocity spectrum in the right common carotid artery before **(A)**, during **(B)**, and immediately after EECP **(C)**.

**FIGURE 2:**
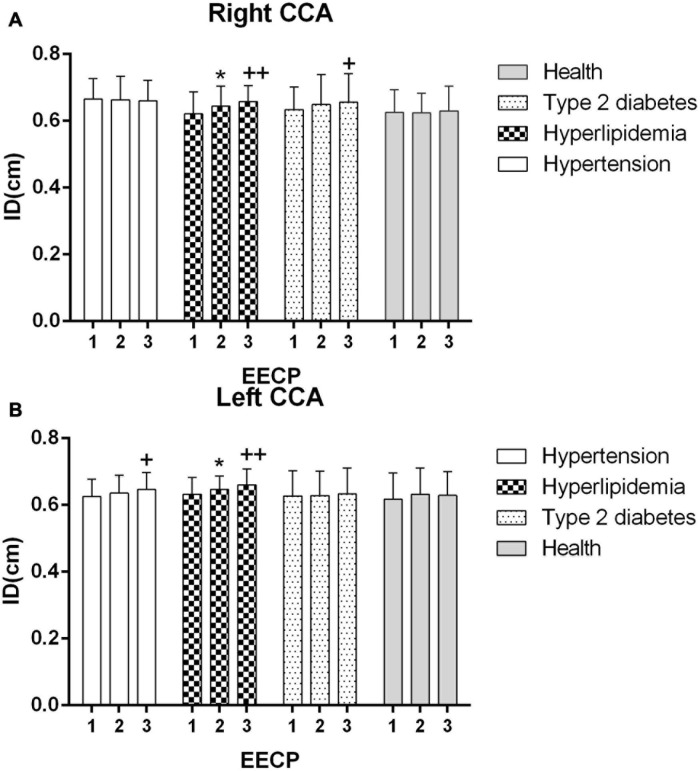
Averaged inner diameters (IDs) in the right **(A)** and left **(B)** common carotid artery (CCA) in four groups before, during and after 45-min EECP. *P < 0.05 during EECP vs. pre-EECP, **P < 0.01; ^ + +^ *P* < 0.05 post-EECP vs. pre EECP, ^ + +^ *P* < 0.01.

**FIGURE 3:**
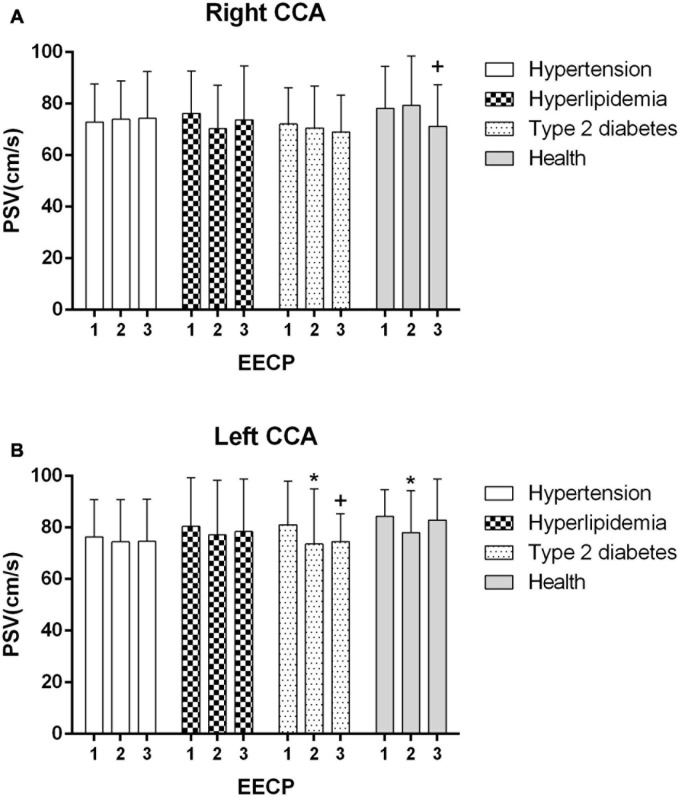
Peak systolic velocity (PSV) in the right **(A)** and left **(B)** common carotid artery (CCA) in four groups before, during and after 45-min EECP. *P < 0.05 during EECP vs. pre-EECP; ^+^*P* < 0.05 post-EECP vs. pre-EECP.

**FIGURE 4:**
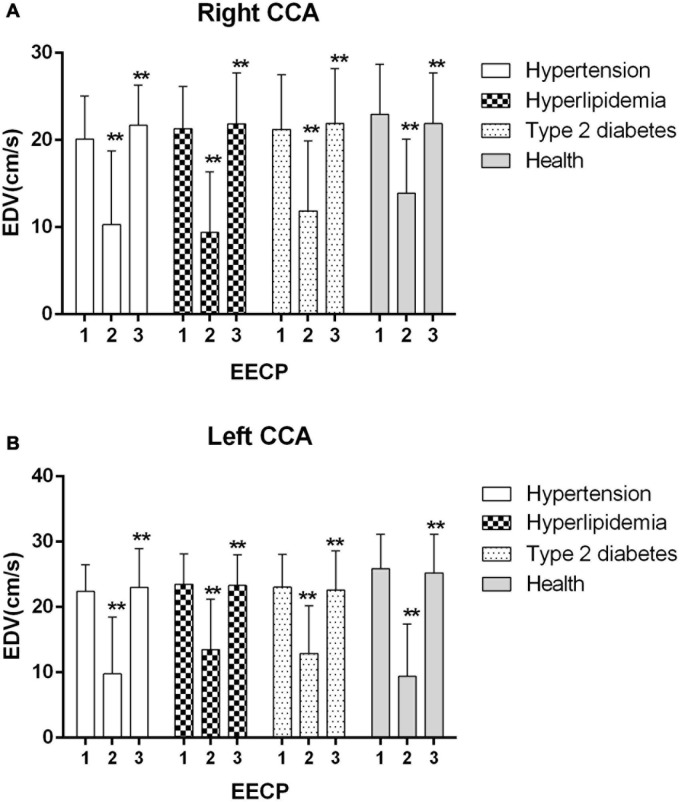
Effect of EECP on the end-diastolic velocity (EDV) of the right **(A)** and left **(B)** common carotid artery (CCA) in four groups before, during and after 45-min EECP. **P < 0.01 during EECP vs. pre-EECP.

**FIGURE 5:**
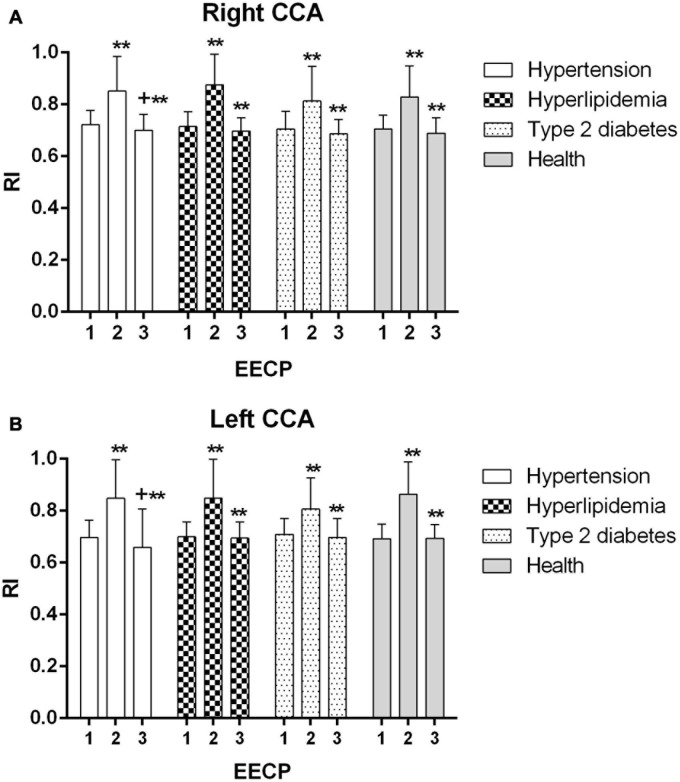
Resistance index (RI) of the right **(A)** and left **(B)** common carotid artery (CCA) in four groups before, during and after 45-min EECP. **P < 0.01 during EECP vs. pre-EECP; ^+^*P* < 0.05 post-EECP vs. pre-EECP.

**FIGURE 6:**
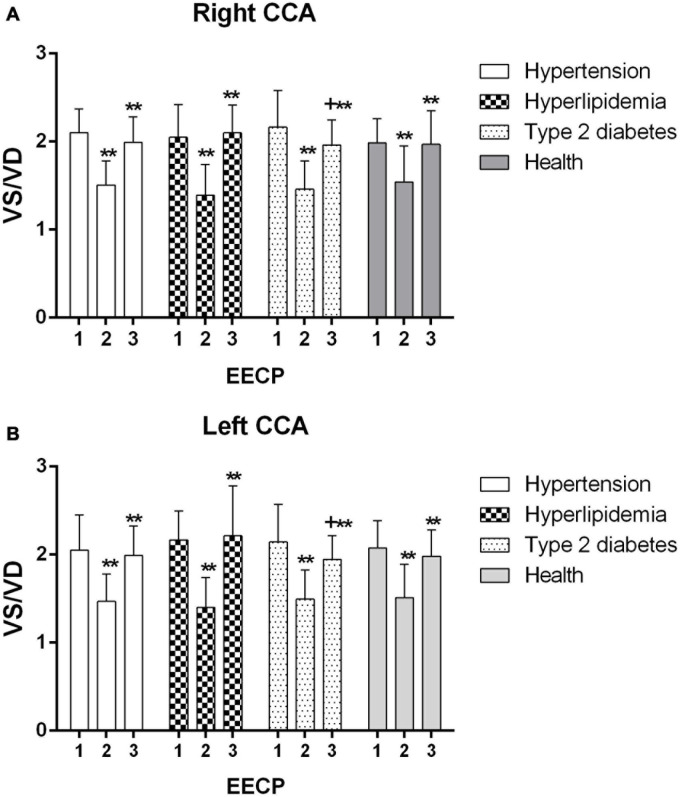
VS/VD of the right **(A)** and left **(B)** common carotid artery (CCA) in four groups before, during and after 45-min EECP. **P < 0.01 during EECP vs. pre-EECP; ^+^*P* < 0.05 post-EECP vs. pre-EECP.

**FIGURE 7:**
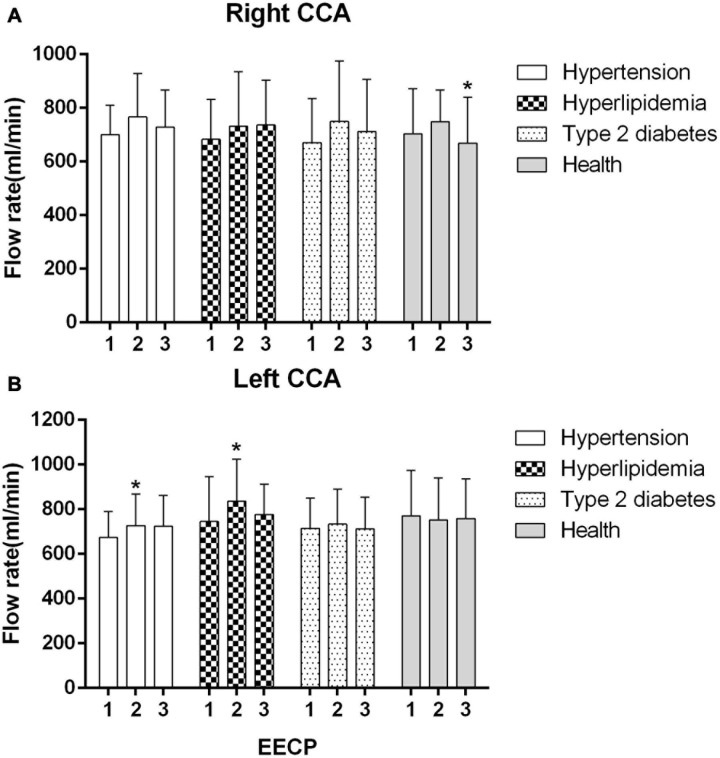
Mean flow rate (FR) of the four groups before, during and after 45-min EECP. *P < 0.05 during EECP vs. pre-EECP.

Ultrasound pictures and Doppler spectrum of right CCA before, during, and immediately after EECP are presented in Figure 1. The effect of EECP on the hemodynamic variables varied in CCAs as did the differences among the four groups. The results are illustrated in Figures 2–7 and summarized in S1 Table 2, which compares the effects of EECP for CCAs in each group separately, and Table 3, which allows multiple comparisons among the four groups.

**TABLE 2:** Hemodynamic variables in the CCAs before, during and immediately after EECP in each group.

**Part**	**Variable**	**EECP**	**Hypertension (*n* = 21)**	***P*-value**	**Hyperlipidemia (*n* = 23)**	***P*-value**	**Type 2 diabetes (*n* = 18)**	***P*-value**	**Controls (*n* = 21)**	***P*-value**
RC	ID	Before	0.666 ± 0.061		0.621 ± 0.066	0.019	0.634 ± 0.067		0.625 ± 0.069	
		During	0.663 ± 0.070		0.644 ± 0.060		0.649 ± 0.09		0.624 ± 0.059	
		After	0.660 ± 0.062		0.658 ± 0.048	0.000	0.656 ± 0.085	0.038	0.63 ± 0.074	
	PSV	Before	72.816 ± 14.801		76.216 ± 16.428		72.136 ± 13.955		78.203 ± 16.234	
		During	73.943 ± 14.887		70.34 ± 16.819		70.546 ± 16.256		79.382 ± 19.123	
		After	74.270 ± 18.24		73.729 ± 21.016		69.014 ± 14.323		71.217 ± 16.056	0.019
	EDV	Before	20.077 ± 4.983	0.000	21.321 ± 4.831	0.000	21.2 ± 6.275	0.001	22.94 ± 5.773	0.000
		During	10.276 ± 8.453	0.000	9.39 ± 6.965	0.000	11.852 ± 8.051	0.000	13.905 ± 6.189	0.000
		After	21.708 ± 4.605		21.857 ± 5.847		21.889 ± 6.294		21.895 ± 5.796	
	RI	Before	0.721 ± 0.551	0.000	0.715 ± 0.057	0.000	0.705 ± 0.068	0.000	0.705 ± 0.053	0.000
		During	0.851 ± 0.134	0.000	0.875 ± 0.119	0.000	0.813 ± 0.133	0.000	0.829 ± 0.120	0.000
		After	0.7 ± 0.614	0.039	0.697 ± 0.051		0.686 ± 0.056		0.689 ± 0.059	
	VS/VD	Before	2.099 ± 0.271	0.000	2.05 ± 0.368	0.000	2.165 ± 0.416	0.000	1.987 ± 0.275	0.000
		During	1.506 ± 0.274	0.000	1.39 ± 0.35	0.000	1.459 ± 0.319	0.000	1.541 ± 0.408	0.000
		After	1.99 ± 0.29		2.10 ± 0.316		1.962 ± 0.285	0.026	1.968 ± 0.382	
	FR	Before	700.381 ± 110.343		682.831 ± 148.092		670.356 ± 163.890	0.049	703.709 ± 167.713	
		During	766.445 ± 161.586		732.16 ± 202.638		749.64 ± 225.135		747.538 ± 118.619	0.039
		After	728.576 ± 138.115		736.31 ± 167.714		711.346 ± 195.599		668.411 ± 171.023	
LC	ID	Before	0.626 ± 0.051		0.632 ± 0.05	0.036	0.627 ± 0.076		0.617 ± 0.079	
		During	0.636 ± 0.053		0.647 ± 0.04		0.628 ± 0.073		0.632 ± 0.079	
		After	0.646 ± 0.051	0.027	0.66 ± 0.048	0.001	0.634 ± 0.077		0.629 ± 0.071	
	PSV	Before	76.335 ± 14.528		80.45 ± 18.933		81.023 ± 16.954	0.043	84.29 ± 10.339	0.048
		During	74.483 ± 16.344		77.126 ± 21.259		73.608 ± 21.309		77.997 ± 16.346	
		After	74.699 ± 16.290		78.444 ± 20.471		74.494 ± 10.838	0.022	82.878 ± 15.877	
	EDV	Before	22.415 ± 4.021	0.000	23.486 ± 4.617	0.000	23.055 ± 5.041	0.000	25.867 ± 5.269	0.000
		During	9.797 ± 8.646	0.000	13.463 ± 7.734	0.000	12.876 ± 7.325	0.000	9.424 ± 7.966	0.000
		After	22.974 ± 5.938		23.315 ± 4.666		22.594 ± 5.996		25.217 ± 5.891	
	RI	Before	0.696 ± 0.068	0.000	0.7 ± 0.057	0.000	0.709 ± 0.061	0.000	0.692 ± 0.057	0.000
		During	0.848 ± 0.149	0.000	0.848 ± 0.15	0.000	0.806 ± 0.121	0.000	0.863 ± 0.126	0.000
		After	0.658 ± 0.149	0.044	0.695 ± 0.062		0.696 ± 0.074		0.694 ± 0.052	
	VS/VD	Before	2.049 ± 0.399	0.000	2.167 ± 0.326	0.000	2.144 ± 0.427	0.000	2.076 ± 0.31	0.000
		During	1.469 ± 0.311	0.000	1.398 ± 0.341	0.000	1.493 ± 0.332	0.000	1.51 ± 0.38	0.000
		After	1.99 ± 0.333		2.213 ± 0.568		1.945 ± 0.271	0.047	1.982 ± 0.298	
	FR	Before	673.992 ± 115.073	0.046	745.701 ± 201.169	0.005	714.45 ± 135.201		770.708 ± 202.409	
		During	726.992 ± 141.483		835.49 ± 188.825		733.362 ± 155.955		751.516 ± 188.786	
		After	723.051 ± 138.335		776.145 ± 135.881		711.132 ± 142.346		758.736 ± 177.745	

*RC and LC are the right carotid and left carotid arteries, respectively. The letters in the p-value column indicate significant differences between variables measured before and during EECP; between during and after EECP; and between before and immediately after EECP.*

**TABLE 3:** Differences of Hemodynamic variables in the common carotid artery among four groups before, during and immediately after 45 min-EECP.

**Label**	**Groups**	**Variables**		***P*-value**		***P*-value**		***P*-value**
		**RC-ID**	**Before**		**During**		**After**	
1	Hypertension (*n* = 21)		0.666 ± 0.061		0.663 ± 0.07		0.66 ± 0.062	
2	Hyperlipidemia (*n* = 23)		0.621 ± 0.066	0.026 (1–2)	0.644 ± 0.06		0.658 ± 0.048	
3	Type 2 diabetes (*n* = 18)		0.634 ± 0.067		0.649 ± 0.09		0.656 ± 0.085	
4	Healthy controls (*n* = 21)		0.625 ± 0.069	0.043 (1–4)	0.624 ± 0.059		0.63 ± 0.074	

		**PSV**	**Before**		**During**		**After**	

1	Hypertension (*n* = 21)		72.816 ± 14.801		73.943 ± 14.887		74.270 ± 18.24	
2	Hyperlipidemia (*n* = 23)		76.216 ± 16.428		70.34 ± 16.819		73.729 ± 21.016	
3	Type 2 diabetes (*n* = 18)		72.136 ± 13.955		70.546 ± 16.256		69.014 ± 14.323	
4	Healthy controls (*n* = 21)		78.203 ± 16.234		79.382 ± 19.123		71.217 ± 16.056	

		**EDV**	**Before**		**During**		**After**	

1	Hypertension (*n* = 21)		20.077 ± 4.983		10.276 ± 8.453		21.708 ± 4.605	
2	Hyperlipidemia (*n* = 23)		21.321 ± 4.831		9.39 ± 6.965		21.857 ± 5.847	
3	Type 2 diabetes (*n* = 18)		21.2 ± 6.275		11.852 ± 8.051		21.889 ± 6.294	
4	Healthy controls (*n* = 21)		22.94 ± 5.773		13.905 ± 6.189	0.048 (2–4)	21.895 ± 5.796	

		**RI**	**Before**		**During**		**After**	

1	Hypertension (*n* = 21)		0.721 ± 0.551		0.851 ± 0.134		0.7 ± 0.614	
2	Hyperlipidemia (*n* = 23)		0.715 ± 0.057		0.875 ± 0.119		0.697 ± 0.051	
3	Type 2 diabetes (*n* = 18)		0.705 ± 0.068		0.813 ± 0.133		0.686 ± 0.056	
4	Healthy controls (*n* = 21)		0.705 ± 0.053		0.829 ± 0.120		0.689 ± 0.059	

		**VS/VD**	**Before**		**During**		**After**	

1	Hypertension (*n* = 21)		2.099 ± 0.271		1.506 ± 0.274		1.99 ± 0.29	
2	Hyperlipidemia (*n* = 23)		2.05 ± 0.368		1.39 ± 0.35		2.10 ± 0.316	
3	Type 2 diabetes (*n* = 18)		2.165 ± 0.416		1.459 ± 0.319		1.962 ± 0.285	
4	Healthy controls (*n* = 21)		1.987 ± 0.275		1.541 ± 0.408		1.968 ± 0.382	

		**FR**	**Before**		**During**		**After**	

1	Hypertension (*n* = 21)		700.381 ± 110.343		766.445 ± 161.586		728.576 ± 138.115	
2	Hyperlipidemia (*n* = 23)		682.831 ± 148.092		732.16 ± 202.638		736.31 ± 167.714	
3	Type 2 diabetes (*n* = 18)		670.356 ± 163.890		749.64 ± 225.135		711.346 ± 195.599	
4	Healthy controls (*n* = 21)		703.709 ± 167.713		747.538 ± 118.619		668.411 ± 171.023	

		**LC-ID**	**Before**		**During**		**After**	

1	Hypertension (*n* = 21)		0.666 ± 0.061		0.663 ± 0.07		0.66 ± 0.062	
2	Hyperlipidemia (*n* = 23)		0.621 ± 0.066		0.644 ± 0.06		0.658 ± 0.048	
3	Type 2 diabetes (*n* = 18)		0.634 ± 0.067		0.649 ± 0.09		0.656 ± 0.085	
4	Healthy controls (*n* = 21)		0.625 ± 0.069		0.624 ± 0.059		0.63 ± 0.074	

		**PSV**	**Before**		**During**		**After**	

1	Hypertension (*n* = 21)		72.816 ± 14.801		73.943 ± 14.887		74.270 ± 18.24	
2	Hyperlipidemia (*n* = 23)		76.216 ± 16.428		70.34 ± 16.819		73.729 ± 21.016	
3	Type 2 diabetes (*n* = 18)		72.136 ± 13.955		70.546 ± 16.256		69.014 ± 14.323	
4	Healthy controls (*n* = 21)		78.203 ± 16.234		79.382 ± 19.123		71.217 ± 16.056	

		**EDV**	**Before**		**During**		**After**	

1	Hypertension (*n* = 21)		20.077 ± 4.983		10.276 ± 8.453		21.708 ± 4.605	
2	Hyperlipidemia (*n* = 23)		21.321 ± 4.831		9.39 ± 6.965		21.857 ± 5.847	
3	Type 2 diabetes (*n* = 18)		21.2 ± 6.275		11.852 ± 8.051		21.889 ± 6.294	
4	Healthy controls (*n* = 21)		22.94 ± 5.773	0.021 (1–4)	13.905 ± 6.189		21.895 ± 5.796	

		**RI**	**Before**		**During**		**After**	

1	Hypertension (*n* = 21)		0.721 ± 0.551		0.851 ± 0.134		0.7 ± 0.614	
2	Hyperlipidemia (*n* = 23)		0.715 ± 0.057		0.875 ± 0.119		0.697 ± 0.051	
3	Type 2 diabetes (*n* = 18)		0.705 ± 0.068		0.813 ± 0.133		0.686 ± 0.056	
4	Healthy controls (*n* = 21)		0.705 ± 0.053		0.829 ± 0.120		0.689 ± 0.059	

		**VS/VD**	**Before**		**During**		**After**	

1	Hypertension (*n* = 21)		2.099 ± 0.271		1.506 ± 0.274		1.99 ± 0.29	
2	Hyperlipidemia (*n* = 23)		2.05 ± 0.368		1.39 ± 0.35		2.10 ± 0.316	
3	Type 2 diabetes (*n* = 18)		2.165 ± 0.416		1.459 ± 0.319		1.962 ± 0.285	0.034 (2–3)
4	Healthy controls (*n* = 21)		1.987 ± 0.275		1.541 ± 0.408		1.968 ± 0.382	

		**FR**	**Before**		**During**		**After**	

1	Hypertension (*n* = 21)		700.381 ± 110.343		766.445 ± 161.586		728.576 ± 138.115	
2	Hyperlipidemia (*n* = 23)		682.831 ± 148.092		732.16 ± 202.638	0.039 (1–2)	736.31 ± 167.714	
3	Type 2 diabetes (*n* = 18)		670.356 ± 163.890		749.64 ± 225.135		711.346 ± 195.599	
4	Healthy controls (*n* = 21)		703.709 ± 167.713		747.538 ± 118.619		668.411 ± 171.023	

*RC, LC, RB, and RF are the right carotid, left carotid, brachial and femoral arteries, respectively.*

*PSV, peak systolic velocity; ID, mean inner diameter; FR, mean flow rate; EDV, end-diastolic velocity; RI, resistance index.*

### Mean ID

The ID of the right CCA was significantly higher in patients with hypertension at baseline compared with patients with hyperlipidemia and healthy controls (both *P* < 0.05), while it had no significant differences in each group during and immediately after EECP (*P* > 0.05). The ID of CCAs was significantly increased only in patients with hyperlipidemia during EECP, and it was continuously increased in patients with hyperlipidemia and type 2 diabetes (*P* < 0.05, Figure 2). Immediately after EECP, the ID of left CCA was also markedly increased in patients with hypertension (*P* < 0.05, Figure 2B).

### PSV

During EECP, the PSV of the left CCAs was significantly reduced in healthy controls and patients with type 2 diabetes (*P* < 0.05, Figure 3B). Immediately after EECP, PSV of right CCA and left CCA was markedly decreased in healthy controls and patients with type 2 diabetes, respectively (*P* < 0.05, Figure 3).

### EDV

EDV of the left CCA was significantly lower in patients with hypertension at baseline, while during EECP, EDV of the right CCA was markedly lower in patients with type 2 diabetes compared with healthy controls (*P* < 0.05). EDV of the CCAs was significantly reduced during EECP and then increased immediately after EECP in each group (all *P* < 0.01, Figure 4B).

### RI

RI of the CCAs was significantly increased during EECP and then markedly decreased immediately after EECP among four groups (all *P* < 0.01, Figure 5). Only in patients with hypertension was RI significantly reduced immediately after EECP (both *P* < 0.01, Figure 5).

### Ratio of PSV to Diastolic Velocity

VS/VD of the CCAs was significantly decreased during EECP and then markedly increased immediately after EECP among four groups (all *P* < 0.01, Figure 6). Immediately after EECP, VS/VD was significantly higher compared with baseline only in patients with type 2 diabetes (both *P* < 0.01, Figure 6). Additionally, VS/VD of left CCA was lower in patients with type 2 diabetes compared with that in patients with hyperlipidemia immediately after EECP (P < 0.05).

### Mean FR

The FR of the left CCA was significantly increased in patients with hypertension and hyperlipidemia during EECP (both *P* < 0.05, Figure 7B). In addition, FR of right CCA was markedly lower in healthy controls during EECP compared with immediately after EECP (*P* < 0.05, Figure 7A).

## Discussion

The present study was designed to investigate carotid hemodynamic responses during and immediately EECP in patients with different cardiovascular risks. Two interesting findings were summarized: first, EECP creates an acute reduction in EDV and VS/VD and an immediate increase in RI of CCAs among four groups. Additionally, the ID of CCAs and FR of left CCA were increased in patients with hyperlipidemia during EECP. PSV of left CCA was reduced in patients with type 2 diabetes; second, a single 45-min EECP intervention produces acute improvement on the ID of patients with hyperlipidemia, RI of patients with hypertension, and PSV and VS/VD of patients with type 2 diabetes.

Few studies have investigated the acute effect of EECP on these hemodynamic parameters in patients with hyperlipidemia. A study had reported that EECP exerts clear arterial effects on large and small vessels of the carotid circulation in CAD patients (Levenson et al., 2007). Our results are consistent, at least by some parameters, with the study of Levenson et al. (2007) ID, EDV, and FR in the CCAs were significantly improved by 45-min EECP intervention. EECP as well as physical training may also activate catecholamines and/or metabolic vasodilator pathways. In addition, a possible mechanism is the reduction of arterial pressure (Armentano et al., 1991).

Picard et al. found that EECP can reduce BP and improve cardiac fitness and arterial stiffness (Picard et al., 2018). By contrast, a study reported that EECP induced the augmentation in cerebral BP in both stroke patients and control (Werner et al., 2003). In the present study, although there was a negative change in RI and EDV in patients with hypertension during EECP, it was a better response immediately after EECP in comparison with baselines in this study. We also found that only in patients with hypertension was RI reduced immediately after EECP. The reduced RI which is strongly associated with cerebrovascular resistance can predict the degree of atherosclerosis (Frauchiger et al., 2001), which is also beneficial to the increase of blood flow.

It showed that the RI reduced immediately after EECP, which was conducive to increasing the perfusion. This finding was consistent with a study that EECP treatment reduces distal brain resistance and increases cerebral perfusion (Lin et al., 2012). A study found that EECP creates an immediate improvement in carotid mean blood flow (Levenson et al., 2007). The other study also found that cerebral blood flow of ischemic stroke patients was increased during EECP, but it does not change in healthy subjects (Lin et al., 2012). Cerebral autoregulation played a part in guaranteeing the constancy of cerebral perfusion during and immediately after EECP (Markus, 2004).

Mean blood flow was significantly elevated in patients with hyperlipidemia during EECP, but it had no significant change immediately after EECP. It was consistent with the findings of Levenson et al. (2007) showing that EECP increased FR in the carotid artery. The related mechanism was caused by a reduction in the regional vascular resistance which was in turn caused by the tone of the vascular smooth muscle (Lincoln et al., 2001; Rybalkin et al., 2003). The other exploration was shown that EECP may have training effects, such as exercise (Levenson et al., 2007). Moreover, a study reported that regulation of blood flow was related to carotid diameters and blood flow velocity (Levenson et al., 1985). Furthermore, another study found that mean carotid artery blood flow did not change due to the decreased systolic blood flow, although blood flow during diastole was significantly increased during intra-aortic balloon pump (Applebaum et al., 1998).

A study reported that blood flow velocity is associated with the regulation of blood flow (Schmidt-Trucksass et al., 1999). In the present study, PSV was reduced in patients with type 2 diabetes during EECP. However, Werner et al. (2003) reported that cerebral flow velocity in systole was elevated during EECP in both patients and healthy controls. It is attributed to decreased SBP and pronounced vascular autoregulation (Werner et al., 2003). Other studies found that increased carotid circulation was associated with a decrease of arterial stiffness and arterial resistance in the carotid artery, which was decreased by shear stress generated by EECP, arterial pressure, and modulation of smooth muscle (Levenson et al., 2007). A study also suggested that PSV is related to arterial diameters and blood flow, which is used to evaluate atherosclerotic risk factors (Schmidt-Trucksass et al., 1999). In our study, the decreased PSV in healthy subjects during EECP may be caused by a better vascular function response.

Meanwhile, the response of PSV in patients with diabetes influenced the change in the VS/VD during and immediately after EECP. EECP creates an acute reduction in VS (PSV)/VD in patients with type 2 diabetes. During EECP, the counterpulsation wave of EECP treatment was superposed in VD, resulting in the change of VS/VD. Few studies have reported on VS/VD in patients with diabetes during EECP. Martin et al. (2013) found that EECP improved the microvascular function in subjects with abnormal glucose tolerance. Blood flow velocity in diastole of the brachial artery is increased by 132%, which led to the increase in brachial artery wall shear stress (WSS) (Zhang et al., 2007). The acute increase of WSS can cause the production of NO, which plays a critical role in vessel relaxation, while chronic NO production because the increased laminal shear stress may serve as an anti-inflammatory and antiatherogenic molecule (Klein-Nulend et al., 1998). Moreover, eNOS, which is regarded as a rate-limiting enzyme essential for NO synthesis and with shear stress-responsive elements in its gene promotor region, may serve as a mechanosensory coupling NO release to hemodynamic responses (Rudic et al., 1998).

Some limitations of the present study should be emphasized. Firstly, the sample size of each group is relatively small. Secondly, we just measure the blood flow data of carotid Doppler ultrasound, which are closely related to cardiovascular events. Thirdly, in order to explore the acute responses of hemodynamics, carotid hemodynamic parameters during and immediately EECP are analyzed in this study. Our results need to be further verified for long-term EECP treatment (36-h EECP intervention).

## Conclusion

EECP created an acute reduction in EDV and VS/VD and an immediate increase in the RI of CCAs among the four groups examined in this study. In addition, the ID of CCAs and FR of the left CCA were increased in patients with hyperlipidemia during EECP. PSV of the left CCA was reduced in patients with type 2 diabetes. Moreover, a single 45-min EECP intervention produces immediate improvement on the ID of patients with hyperlipidemia, RI of patients with hypertension, and PSV and VS/VD of patients with type 2 diabetes. These findings indicate that the different hemodynamic parameters induced by EECP are highlighted in different patients. Moreover, EECP can regulate the vascular and blood flow characteristics of carotid arteries and further improve the carotid function in patients with high cardiovascular risk factors.

## Data Availability Statement

The original contributions presented in the study are included in the article/supplementary material, further inquiries can be directed to the corresponding author/s.

## Ethics Statement

The studies involving human participants were reviewed and approved by the Eighth Affiliated Hospital of Sun Yat-sen University. The patients/participants provided their written informed consent to participate in this study. Written informed consent was obtained from the individual(s) for the publication of any potentially identifiable images or data included in this article.

## Author Contributions

YZ and GW proposed the scientific problems. YZ, GW, and JD designed the experiments. YZ, ZM, CY, and WZ collected the experimental data. YZ and ZM processed and calculated the data. YZ conducted the statistical analysis and wrote the draft manuscript. JD, XZ, HH, and GW contributed to the revision and final version of the manuscript. All authors contributed to the article and approved the submitted version.

## Conflict of Interest

The authors declare that the research was conducted in the absence of any commercial or financial relationships that could be construed as a potential conflict of interest.

## References

[B1] Applebaum R. M. Wun H. H. Katz E. S. Tunick P. A. Kronzon I. (1998). Effects of intraaortic balloon counterpulsation on carotid artery blood flow. *Am. Heart J.* 135(Pt 1) 850–854. 10.1016/s0002-8703(98)70045-6 9588416

[B2] Armentano R. Simon A. Levenson J. Chau N. P. Megnien J. L. Pichel R. (1991). Mechanical pressure versus intrinsic effects of hypertension on large arteries in humans. *Hypertension* 18 657–664. 10.1161/01.hyp.18.5.657 1937668

[B3] Bai C. H. Chen J. R. Chiu H. C. Pan W. H. (2007). Lower blood flow velocity, higher resistance index, and larger diameter of extracranial carotid arteries are associated with ischemic stroke independently of carotid atherosclerosis and cardiovascular risk factors. *J. Clin. Ultrasound* 35 322–330. 10.1002/jcu.20351 17471583

[B4] Bondesson S. Pettersson T. Ohlsson O. Hallberg I. R. Wackenfors A. Edvinsson L. (2010). Effects on blood pressure in patients with refractory angina pectoris after enhanced external counterpulsation. *Blood Press.* 19 287–294. 10.3109/08037051003794375 20429696

[B5] Braith R. W. Conti C. R. Nichols W. W. Choi C. Y. Khuddus M. A. Beck D. T. (2010). Enhanced external counterpulsation improves peripheral artery flow-mediated dilation in patients with chronic angina: a randomized sham-controlled study. *Circulation* 122 1612–1620. 10.1161/circulationaha.109.923482 20921442 PMC2963100

[B6] Chuang S. Y. Bai C. H. Cheng H. M. Chen J. R. Yeh W. T. Hsu P. F. (2016). Common carotid artery end-diastolic velocity is independently associated with future cardiovascular events. *Eur. J. Prev. Cardiol.* 23 116–124. 10.1177/2047487315571888 25691545

[B7] Dockery F. Rajkumar C. Bulpitt C. J. Hall R. J. Bagger J. P. (2004). Enhanced external counterpulsation does not alter arterial stiffness in patients with angina. *Clin. Cardiol.* 27 689–692. 10.1002/clc.4960271206 15628111 PMC6654362

[B8] Frauchiger B. Schmid H. P. Roedel C. Moosmann P. Staub D. (2001). Comparison of carotid arterial resistive indices with intima-media thickness as sonographic markers of atherosclerosis. *Stroke* 32 836–841. 10.1161/01.str.32.4.836 11283379

[B9] Gloekler S. Meier P. de Marchi S. F. Rutz T. Traupe T. Rimoldi S. F. (2010). Coronary collateral growth by external counterpulsation: a randomised controlled trial. *Heart* 96 202–207. 10.1136/hrt.2009.184507 19897461

[B10] Gurovich A. N. Braith R. W. (2013). Enhanced external counterpulsation creates acute blood flow patterns responsible for improved flow-mediated dilation in humans. *Hypertens Res* 36 297–305. 10.1038/hr.2012.169 23076403

[B11] Kallikazaros I. Tsioufis C. Sideris S. Stefanadis C. Toutouzas P. (1999). Carotid artery disease as a marker for the presence of severe coronary artery disease in patients evaluated for chest pain. *Stroke* 30 1002–1007. 10.1161/01.str.30.5.1002 10229735

[B12] Klein-Nulend J. Helfrich M. H. Sterck J. G. H. MacPherson H. Joldersma M. Ralston S. H. (1998). Nitric oxide response to shear stress by human bone cell cultures is endothelial nitric oxide synthase dependent. *Biochem. Biophys. Res. Commun.* 250 108–114. 10.1006/bbrc.1998.9270 9735341

[B13] Lee S. W. Hai J. J. Kong S. L. Lam Y. M. Lam S. Chan P. H. (2011). Side differences of carotid intima-media thickness in predicting cardiovascular events among patients with coronary artery disease. *Angiology* 62 231–236. 10.1177/0003319710379109 20688786

[B14] Levenson J. Simon A. Megnien J. L. Chironi G. Gariepy J. Pernollet M. G. (2007). Effects of enhanced external counterpulsation on carotid circulation in patients with coronary artery disease. *Cardiology* 108 104–110. 10.1159/000095949 17008798

[B15] Levenson J. A. Simon A. C. Safar M. E. (1985). Vasodilatation of small and large arteries in hypertension. *J. Cardiovasc. Pharmacol.* 7(Suppl. 2) S115–S120. 2409359 10.1097/00005344-198507002-00022

[B16] Lin W. Xiong L. Han J. Leung T. W. Soo Y. O. Chen X. (2012). External counterpulsation augments blood pressure and cerebral flow velocities in ischemic stroke patients with cerebral intracranial large artery occlusive disease. *Stroke* 43 3007–3011. 10.1161/strokeaha.112.659144 22996956

[B17] Lincoln T. M. Dey N. Sellak H. (2001). Invited review: cGMP-dependent protein kinase signaling mechanisms in smooth muscle: from the regulation of tone to gene expression. *J. Appl. Physiol. (1985)* 91 1421–1430. 10.1152/jappl.2001.91.3.1421 11509544

[B18] Liu H. B. Yuan W. X. Qin K. R. Hou J. (2015). Acute effect of cycling intervention on carotid arterial hemodynamics: basketball athletes versus sedentary controls. *Biomed. Eng. Online* 14(Suppl. 1):S17. 25602805 10.1186/1475-925X-14-S1-S17 PMC4306107

[B19] Loizou C. P. Nicolaides A. Kyriacou E. Georghiou N. Griffin M. Pattichis C. S. (2015). A comparison of ultrasound intima-media thickness measurements of the left and right common carotid artery. *IEEE J. Transl. Eng. Health Med.* 3:1900410. 10.1109/jtehm.2015.2450735 27170894 PMC4848048

[B20] Markus H. S. (2004). Cerebral perfusion and stroke. *J. Neurol. Neurosurg. Psychiatry* 75 353–361. 10.1136/jnnp.2003.025825 14966145 PMC1738983

[B21] Martin J. S. Beck D. T. Braith R. W. (2013). Peripheral resistance artery blood flow in subjects with abnormal glucose tolerance is improved following enhanced external counterpulsation therapy. *Appl. Physiol. Nutr. Metab.* 39 596–599. 10.1139/apnm-2013-0497 24766247

[B22] Masuda D. Nohara R. Hirai T. Kataoka K. Chen L. Hosokawa R. (2001). Enhanced external counterpulsation improved myocardial perfusion and coronary flow reserve in patients with chronic stable angina. Evaluation by13N-ammonia positron emission tomography. *Eur. Heart J.* 22 1451–1458. 10.1053/euhj.2000.2545 11482918

[B23] Michaels A. D. (2002). Left ventricular systolic unloading and augmentation of intracoronary pressure and doppler flow during enhanced external counterpulsation. *Circulation* 106 1237–1242. 10.1161/01.cir.0000028336.95629.b0 12208799

[B24] Nichols W. W. Estrada J. C. Braith R. W. Owens K. Conti C. R. (2006). Enhanced external counterpulsation treatment improves arterial wall properties and wave reflection characteristics in patients with refractory angina. *J. Am. Coll. Cardiol.* 48 1208–1214. 10.1016/j.jacc.2006.04.094 16979007

[B25] Ozari H. O. Oktenli C. Celik S. Tangi F. Ipcioglu O. Terekeci H. M. (2012). Are increased carotid artery pulsatility and resistance indexes early signs of vascular abnormalities in young obese males? *J. Clin. Ultrasound* 40 335–340. 10.1002/jcu.21927 22532370

[B26] Picard F. Panagiotidou P. Wolf-Pütz A. Buschmann I. Buschmann E. Steffen M. (2018). Usefulness of individual shear rate therapy, new treatment option for patients with symptomatic coronary artery disease. *Am. J. Cardiol.* 121 416–422. 10.1016/j.amjcard.2017.11.004 29274808

[B27] Rudic R. D. Shesely E. G. Maeda N. Smithies O. Segal S. S. Sessa W. C. (1998). Direct evidence for the importance of endothelium-derived nitric oxide in vascular remodeling. *J. Clin. Invest.* 101 731–736. 10.1172/jci1699 9466966 PMC508619

[B28] Rybalkin S. D. Yan C. Bornfeldt K. E. Beavo J. A. (2003). Cyclic GMP phosphodiesterases and regulation of smooth muscle function. *Circ. Res.* 93 280–291. 10.1161/01.res.0000087541.15600.2b 12933699

[B29] Sardina P. D. Martin J. S. Dzieza W. K. Braith R. W. (2016). Enhanced external counterpulsation (EECP) decreases advanced glycation end products and proinflammatory cytokines in patients with non-insulin-dependent type II diabetes mellitus for up to 6 months following treatment. *Acta Diabetol.* 53 753–760. 10.1007/s00592-016-0869-6 27278477

[B30] Schmidt-Trucksass A. Grathwohl D. Schmid A. Boragk R. Upmeier C. Keul J. (1999). Structural, functional, and hemodynamic changes of the common carotid artery with age in male subjects. *Arterioscler. Thromb. Vasc. Biol.* 19 1091–1097. 10.1161/01.atv.19.4.1091 10195940

[B31] Sedaghat S. van Sloten T. T. Laurent S. London G. M. Pannier B. Kavousi M. (2018). Common carotid artery diameter and risk of cardiovascular events and mortality: pooled analyses of four cohort studies. *Hypertension* 72 85–92. 10.1161/hypertensionaha.118.11253 29785959

[B32] Werner D. Marthol H. Brown C. M. Daniel W. G. Hilz M. J. (2003). Changes of cerebral blood flow velocities during enhanced external counterpulsation. *Acta Neurol. Scand.* 107 405–411. 10.1034/j.1600-0404.2003.00074.x 12757472

[B33] Zhang Y. He X. Chen X. Ma H. Liu D. Luo J. (2007). Enhanced external counterpulsation inhibits intimal hyperplasia by modifying shear stress responsive gene expression in hypercholesterolemic pigs. *Circulation* 116 526–534. 10.1161/circulationaha.106.647248 17620513

[B34] Zhang Y. Jiang Z. Qi L. Xu L. Sun X. Chu X. (2018). Evaluation of cardiorespiratory function during cardiopulmonary exercise test in untreated hypertensive and healthy subjects. *Front. Physiol.* 9:1590. 10.3389/fphys.2018.01590 30487751 PMC6246679

